# Changes in the Distribution of Cocaine- and Amphetamine-Regulated Transcript-Containing Neural Structures in the Human Colon Affected by the Neoplastic Process

**DOI:** 10.3390/ijms19020414

**Published:** 2018-01-31

**Authors:** Agnieszka Oponowicz, Anna Kozłowska, Sławomir Gonkowski, Janusz Godlewski, Mariusz Majewski

**Affiliations:** 1 Department of Human Physiology, School of Medicine, Collegium Medicum, University of Warmia and Mazury in Olsztyn, ul. Warszawska 30, 10-561 Olsztyn, Poland; kozlowska.anna@uwm.edu.pl (A.K.); mariusz.majewski@uwm.edu.pl (M.M.); 2 Departement of Clinical Physiology, Faculty of Veterinary Medicine, University of Warmia and Mazury in Olsztyn, ul Oczapowskiego 13, 10-718 Olsztyn, Poland; slawomir.gonkowski@uwm.edu.pl; 3 Department of Human Histology and Embryology, School of Medicine, Collegium Medicum, University of Warmia and Mazury in Olsztyn, ul. Warszawska 30, 10-561 Olsztyn, Poland; janusz350@poczta.onet.pl

**Keywords:** cocaine- and amphetamine-regulated transcript, CART, enteric nervous system, colon cancer, carcinoma

## Abstract

The present study analysed changes in the distribution pattern of cocaine- and amphetamine-regulated transcript (CART) in the enteric nervous system (ENS) of the human colon challenged by adenocarcinoma invasion, using the double-labelling immunofluorescence technique. In control specimens, CART immunoreactivity was found in neurons of all studied plexuses, representing 30.1 ± 4.1%, 12.9 ± 5.2%, and 4.1 ± 1.3% of all neurons forming the myenteric plexus (MP), outer submucous plexus (OSP), and inner submucous plexus (ISP), respectively. Tumour growth into the colon wall caused an increase in the relative frequency of CART-like immunoreactive (CART-LI) neurons in enteric plexuses located in the vicinity of the infiltrating neoplasm (to 36.1 ± 6.7%, 32.7 ± 7.3% and 12.1 ± 3.8% of all neurons in MP, OSP and ISP, respectively). The density of CART-LI nerves within particular layers of the intestinal wall did not differ between control and adenocarcinoma-affected areas of the human colon. This is the first detailed description of the CART distribution pattern within the ENS during the adenocarcinoma invasion of the human colon wall. The obtained results suggest that CART probably acts as a neuroprotective factor and may be involved in neuronal plasticity evoked by the progression of a neoplastic process.

## 1. Introduction

It is well known that gastrointestinal (GI) activities are regulated both by the extrinsic innervation and the enteric nervous system (ENS) located in the wall of the GI tract [1,2]. Nerve fibers supplying the stomach and intestinal wall are divided into afferent and efferent (sympathetic and parasympathetic) nerves. The first of them are the processes of sensory neurons located in the dorsal root ganglia or sensory ganglia of the vagus nerve. In turn, sympathetic innervation is derived from neurons located between the Th_8_ and L_2_ neuromers of the spinal cord, while parasympathetic innervation is represented by the vagus nerve and nerves originating from the S_3_ to S_4_ segments of the spinal cord. Sympathetic nerves inhibit GI motility and secretion, and cause the sphincter and blood vessel to contract, whereas parasympathetic nerves stimulate these functions [3,4].

The ENS, consisting of millions of cells, is a part of the autonomic nervous system that integrates all GI functions, and its construction depends on the animal species and fragment of the GI tract. The human ENS in the small and large intestines is built up of four plexuses: the myenteric plexus (MP), located between longitudinal (LM) and circular muscle (CM) layers; the outer submucous plexus (OSP) near the inner side of CM; the inner submucous plexus (ISP), situated between the muscularis mucosa (MM) and the lamina propria; and the intermediate submucous plexus (IMSP), an aganglionated nerve plexus between the OSP and the ISP (closer to the ISP) [5,6]. Each “kind” of plexus coordinates different functions of the human intestine: the MP is responsible for muscle contractions and peristalsis, and the ISP mainly regulates intestinal secretion, ion absorption, and blood vessel contraction—whereas the OSP plays important roles in the control of intestinal motility and ion transport. The functions of the IMSP have not yet been fully explained, but this plexus in the human colon, in respect to its neurochemical content, is approximated to the OSP [5,7].

All plexuses are connected through numerous nerve fibers supplying particular layers of the intestine wall. Neurons of the ENS are very manifold in terms of their conformation, functions, electrophysiological properties, and neurochemical coding, and can be classified according to these properties into different types [8,9,10]. The most diverse and clearest is classification of enteric neurons, in view of their neurochemical coding.

Besides acetylcholine (which is the classic main transmitter in the ENS), both cell bodies located in the enteric plexuses, as well as the nerve fibers connecting them, use a broad spectrum of other substances, which can function as neuromediators or neuromodulators [8]. One of them is cocaine- and amphetamine-regulated transcript peptide (CART). This peptide was identified for the first time by Spiess et al. [11] in the ovine hypothalamus. CART is a regulatory peptide that can be produced in the central nervous system [12] and peripheral sympathetic, sensory [13] or enteric neurons [14], as well as by neuroendocrine cells, including the pancreatic islets cells [15], the mucosal layer of the GI tract [16], as well as the thyroid and adrenal medulla [17]. Thus, it appears that CART acts both as a neurotransmitter and as a hormone. However, the exact functions of CART in the GI tract remain unclear [18]. Peripheral CART-induced effects, i.e., regulation of the contractions of intestinal muscles, may result from its central action [19]. Previous studies on the GI tract have described the numerous CART-immunoreactive neurons and nerve fibers in the MP and CM [20,21,22], which may suggest that CART is involved in the regulation of the intestinal motility and secretion.

It is relatively well-known that the ENS in the large intestine can undergo changes during the course of many diseases. Nerve damage within the large intestine has been observed during inflammatory diseases [23,24], as well as in colon cancer [25]. Moreover, in the case of the latter disease, the most visible changes concerned the expression of neuronal active substances in the ENS [26,27,28,29]. Previous studies showed that CART-LI in the enteric neurons may also be subject to such changes.

A particularly significant increase in the number of CART-positive nerve fibers within the mucosal layer of the descending colon was found in patients with ulcerative colitis [30]. CART is also produced in the majority of neuroendocrine tumours, regardless of tumour origin. CART-expressing tumour cells were found in gastric, ileal, or rectal carcinoids [31]. Recent studies show that CART expression in small bowel carcinoid tumours is associated with lower rates of patient survival [32]. These findings suggest the participation of CART in neuroprotective processes within the ENS and in conduction of sensory stimuli. It is known that functions of neurons in the ENS depend on their neurochemical characterisation, which undergoes pronounced plastic changes under pathological conditions; however, there is still sparse knowledge on CART distribution and its functions in the human GI tract when affected by a tumour. Therefore, the first aim of this study was to determine the distribution of CART-positive neurons in large intestinal submucosal and myenteric plexuses, in the vicinity of the tumour infiltration. The second aim was to investigate whether the number of CART-positive nerve fibers supplying the muscle layers of the intestine wall in healthy tissue differs from those observed in tumour-amended tissue.

## 2. Results

### 2.1. Neurochemical Phenotype of Neurons from Studied Myenteric and Submucous Plexuses

**Figure 1:**
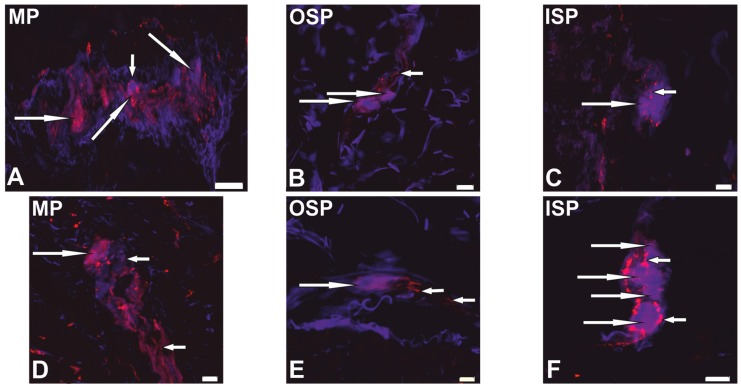
Distribution pattern of neurons (long arrows) and nerve fibers (short arrows) immunoreactive to protein gene-product 9.5 (PGP 9.5, blue) and cocaine- and amphetamine-regulated transcript (CART, red) within the myenteric plexus (MP), outer submucous plexus (OSP), and inner submucous (ISP) plexus of the control (**A**–**C**) and cancer-affected (**D**–**F**) human colonic wall. Figures (**A**–**F**) show the overlap of both stainings. Scale bar = 20 µm.

**Figure 2:**
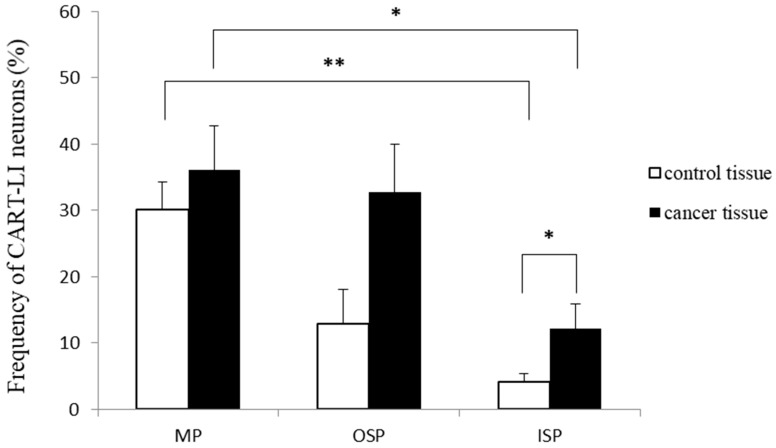
The mean percentage of CART-like immunoreactive (CART-LI) neurons in the myenteric plexus (MP), outer submucosal plexus (OSP), and inner submucosal plexus (ISP) in control and cancer-affected areas of the human colonic wall. Data are presented as means ± SEM; * *p* < 0.05, ** *p* < 0.01.

The presence of CART-LI was found in nerve cell bodies of all types of ganglionated plexuses studied (MP, OSP and ISP), derived from both the control tissue and the cancer-affected region (Figure 1). In the control intestine, the percentage of CART-positive neurons clearly depended on the “kind” of plexus. The highest percentage of such neurons (in relation to all cells immunostained for protein gene product (PGP) 9.5) was noted in the MP (30.1 ± 4.1%), while in the OSP and ISP the CART-LI neuronal cells were less numerous (12.9 ± 5.2% and 4.1 ± 1.3%, respectively; Figure 2). Moreover, neoplastic infiltration caused a three-fold increase (*p* < 0.05) in the percentage of CART-positive neurons within the ISP (from 4.1 ± 1.3% to 12.1 ± 3.8%) when compared to the control tissue (Figure 2). Additionally, the percentage of nerve cells bodies expressing immunoreactivity to CART in both control and cancer tissue was significantly higher (*p* < 0.05–0.01) in the MP than in the ISP. However, differences in the number of cell bodies containing this peptide in the MP (from 30.1 ± 4.2% to 36.1 ± 6.7%) and the OSP (from 12.9 ± 5.2% to 32.7 ± 7.3%), between surgical margin and neoplasmatic regions, were found to be not statistically different (Figure 2). In a solid tumour, the ENS structures were not observed. Furthermore, the presence of CART-positive nerve fibers was observed within all “kinds” of plexuses, in both control and carcinoma-affected tissues.

The total number of PGP 9.5-immunoreactive neuronal cells bodies forming each “kind” of studied plexus did not differ between control and carcinoma affected tissues (Table 1). It is worth adding that in control tissue, the total number of neurons in the MP was significantly higher compared to the OSP and ISP, while in the cancer tissue these differences were not found.

### 2.2. Distribution Patterns of Nerve Fibers Containing CART in Muscle Layers of the Colonic Wall

**Figure 3:**
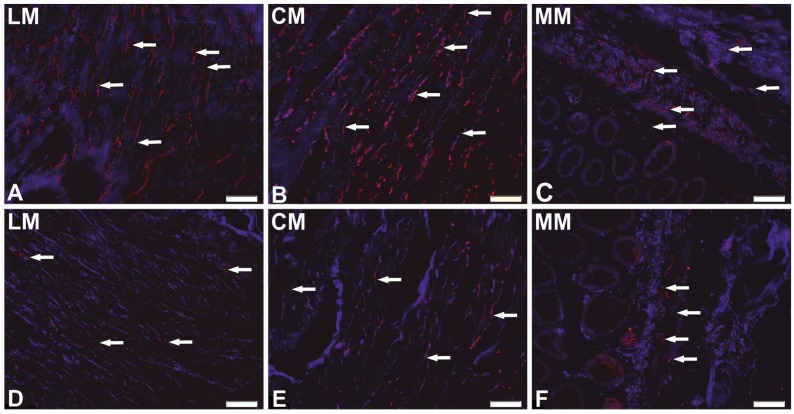
Distribution pattern of nerve fibers (arrows) immunoreactive to CART within longitudinal muscle (LM) and circular muscle (CM) layers, as well as lamina muscularic mucosa (MM), in control (**A**–**C**) and cancer-affected (**D**–**F**) areas of the human colonic wall. Scale bar = 100 µm.

Analyses of nerve fibers containing CART revealed their presence in all muscle layers of the colonic wall: lamina muscularis mucosa (MM), circular muscle (CM), and longitudinal muscle (LM) layers derived from both the sites of cancer invasion and in unchanged colonic fragments (Figure 3).

There were no differences between control and cancer-amended tissues in both the number and morphology of such nerve fibers (Table 2). However, in the control tissue, the least nerve fibers per area unit (*p* < 0.05) were found in LM (0.011 ± 0.002/µm^2^) in comparison both to CM (0.031 ± 0.004/µm^2^) and MM (0.046 ± 0.014/µm^2^). Changes in the density of muscular innervation were observed in carcinoma-affected tissue, where the number of nerve fibers in LM (0.014 ± 0.002/µm^2^) was significantly smaller (*p* < 0.01) in comparison to CM (0.036 ± 0.006/µm^2^), but not to MM (0.034 ± 0.008/µm^2^, *p* = 0.08). 

## 3. Discussion

This is the first report describing (1) the presence of CART in neurons forming ganglionated plexuses (the MP, OSP, and ISP), and (2) the density of nerve fibres containing this peptide in the muscle layers of the colonic wall, in both control tissue derived from the operative margin and in tissue affected by cancer. Until now, the presence of CART has been described in the ENS of healthy humans [18] and pigs [33].

In addition, the present study also indicates that the differences in the percentage of neurons containing CART in the control and cancer-affected tissue depends on the kind of enteric plexuses. For example, the percentage of neurons containing this peptide in the MP was always significantly higher compared to the ISP. It should be pointed out that there is a lack of data concerning the distribution of CART in both healthy and neoplastic-process-affected human colons. However, it was reported in pigs (useful as an animal model in biomedical research [34]) that under physiological conditions, the percentage of CART-LI neurons in the MP within the small intestine ranges from about 10% in the duodenum to above 20% in the ileum [35], and in the large intestine did not exceed 8% [36]. However, the percentage of these enteric neurons in submucosal plexuses was clearly lower than that observed in the MP, and was usually not higher than 5% of all neuronal cells [35,36]. The partial discrepancy between the present results (the percentage of CART-LI neurons), Wojtkiewicz et al. [35], and Gonkowski et al. [36] may be caused by species-specific differences [37].

During previous investigations, CART has been described not only in enteric neurons, but also within intramural nerves in the GI tract. For example, Wierup et al. [17] found an abundant network of fibers immunoreactive to this peptide in many parts of the human GI tract, where the most numerous CART-positive nerve fibers were located in external muscle layers, slightly lower in the intestinal submucosa, and rather sparsely in the mucosa. Moreover, those authors noted that the density of intestinal CART-positive nerves was higher in the distal parts of the GI tract. In the present study, it was observed that in the control tissue, the majority of CART-positive nerve fibers were present in the MM when compared to the LM and CM. However, these data appear to be somewhat contradictory with the results of other authors, who reported that a large number of CART-positive nerve fibres were observed in the circular muscle layer of the human descending colon and caecum [20,21,22]. On the other hand, the results from the current paper for regions affected by cancer invasion corresponds well with those results. The discrepancies may be attributed to the differences in the studied sections of the digestive tract and/or pathological stage.

The present findings clearly show that cancer may change the number of CART-LI enteric neurons. It is in agreement with previous observations, which confirm modifications in the expression of neuronal active substances by enteric neurons during various intestinal and extra-intestinal pathological states, such as inflammatory processes [38], neuronal damage [39], diabetes [40], or the influence of toxins in the food [41]. One of the pathological processes which can affect the neurochemical coding of enteric neurons is neoplastic proliferation in the GI tract. Godlewski [25] has observed the alterations in the structure and localization of enteric plexuses close to the tumour in the human colon, and these changes involved the disappearance of enteric neurons and nerve fibres forming these plexuses in the overall atrophy of enteric nervous structures. In solid tumours, structures of the ENS have not been observed. The present observations also confirm the plasticity of the ENS during neoplasmatic proliferation of the large human intestine.

Additionally, some studies describe that modifications in CART-LI in the human ENS, under the influences of various intra- and extra-intestinal pathological processes (and the character of these changes), clearly depend on the type of acting factor. For example, an increase in the expression of CART within the intestinal nervous structures has been observed during ulcerative colitis [30]. In contrast, a decrease in the number of enteric nervous structures immunoreactive to CART was noted as an effect of Hirschsprung’s disease [42]. However, the increase in the number of CART-LI enteric nervous structures observed in the present study is in accordance with the results of previous studies on neuroendocrine tumours, which (regardless of the tumour origin or stage) also produce changes in the expression of CART [31,43].

It should be noted that knowledge of the functions of CART within the ENS is lacking, contrary to the central nervous system, where this peptide is considered to be one of the most important substances regulating the functions of satiation and hunger centres [19,44]. It is known that CART within the GI tract takes part in the stimulation of colonic muscles [45] and reduces gastric acid secretion [46], but the mechanisms of these activities are unknown. The distribution of CART-positive neuronal cells in all “kinds” of enteric plexuses, observed in both the present study and in previous investigations [16,33,37] suggests that this peptide participates in various aspects of intestinal physiology, such as muscular and secretory activity, as well as intestinal blood flow and ion transport. However, the highest number of neuronal structures immunoreactive to CART localized in the MP and circular muscle layer may indicate that the main role of this peptide in the GI tract is participation in regulatory processes associated with intestinal motility. However, further studies are necessary to confirm this hypothesis.

Moreover, previous studies which described the influence of various diseases on the expression of CART in the ENS [30,31,47,48] strongly suggest adaptive or neuroprotective roles of this peptide in pathologically-affected intestines. The present study shows that one of pathological states during which CART can play the roles mentioned above is colon cancer. It should be noted that the exact mechanisms of observed changes are difficult to explain, due to the fact that functions of CART in the GI tract are not fully understood. It may be connected with direct damage to enteric neurons by neoplastic proliferation, pain stimuli conduction, or derivative disturbances in intestinal motility and excretive activity. In addition, the observed modification may result from various mechanisms, such as the augmentation of CART synthesis (which can arise from changes in transcription, translation, post-translational modifications, or disturbances in the activity of enzymes included in CART synthesis) or modifications to the neuronal transport of CART from cell bodies to nerve endings. However, this hypothesis needs to be further verified in detail.

However, the most likely explanation of changes in CART-LI observed during the present study is the participation of this peptide in neuroprotective processes in the ENS. This is supported by previous studies on other parts of the nervous system. In particular, it is known that CART can act as an endogenous antioxidant [49] and participates in neurotrophic and neuronal regenerative processes [47,50]; it also may be involved in the development of the nervous system [51].

In conclusion, the present results show that CART is widespread in neuronal structures of the ENS in the human large intestine, which may suggest that this peptide is an important neuromediator or neuromodulator involved in the regulation of the GI tract functions. Moreover, a clear increase in CART-like immunoreactivity in neuronal cell bodies and nerve fibers in all parts of the colonic ENS during neoplastic proliferation may indicate the neuroprotective role of this peptide, and its involvement in neuronal plasticity and neuroprotective processes within the ENS. Taken together, CART potentially plays multiple functions in the large human intestine, both in physiological conditions and during carcinoma infiltration, but many aspects of its activity within the GI tract remain unknown and need further investigation.

## 4. Materials and Methods

### 4.1. Patient Recruitment

The present study was conducted using post-operative material derived from eight patients (four men and four women) with diagnosed cancer of the sigmoid colon (CRC), which was collected during surgery at Department of Oncological Surgery of the Regional Oncological Centre in Olsztyn (Poland). The mean age of the patients was 68.1 ± 9.7 years (range 52–78 years). The pathomorphological analyses of the tissues obtained during surgical operations confirmed that all patients included to this study represented a homogenous group. They suffered from the same degree of adenocarcinoma invasion within colonic wall, defined as T3 in the TNM Classification of Malignant Tumors by the American Joint Committee on Cancer (AJCC). The protocol of this study was approved by the University of Warmia and Mazury Bioethics Commission (No. 18/2012, 29 November 2012).

### 4.2. Tissue Preparation

Directly after intestine resection during the surgery, small samples (1 cm × 1 cm) of the intestinal wall were collected for immunohistochemical analyses. Fragments of the colonic wall were taken from the region of cancer invasion and from cancer-unaffected regions at least 5 cm away from the tumour. The samples were fixed by immersion in 4% neutral buffered formaldehyde (pH 7.4) for 120 min, washed in 0.1 M phosphate buffer, and stored in 18% sucrose for 7 days. After that time, the samples were frozen and stored (−20 °C) until sectioning.

### 4.3. Double-Labelling Immunofluorescence

The samples of the intestinal wall were cut into 10-µm-thick cryostat (Thermoscientific Microm HM 525, Waltham, MA, USA) sections for immunofluorescence staining. The sections were air-dried at room temperature (RT) for 45 min and rinsed (3 × 15 min) with phosphate buffered saline (PBS, pH 7.4). The sections were then blocked for 1 h at RT with a blocking buffer containing 1% Triton X100 (Sigma-Aldrich, St. Louis, MO, USA), 0.1% bovine serum albumin (Sigma-Aldrich, St. Louis, MO, USA), 0.05% thimerosal (Sigma-Aldrich, St. Louis, MO, USA), 0.01% NaN_3_ (POCH, Gliwice, Poland), and 10% normal goat serum (Jackson Immunoresearch, West Grove, PA, USA) in 0.01 M PBS, to reduce non-specific background staining. After a triple rinse (3 × 15 min) with PBS, sections were incubated with a mixture of primary antibodies (derived from a different species) against CART and PGP 9.5 overnight in a humid chamber at RT. After the incubation, sections were rinsed with PBS (3 × 15 min) and incubated for 1 h with biotynylated goat anti-rabbit immunoglobulin G (IgG). Following subsequent rising in PBS (3 × 15 min), the sections were incubated (1 h) with a mixture of aminomethylcoumarin (AMCA)-conjugated, donkey, anti-mouse IgG-specific antisera, and streptavidin-conjugated, cyanine 3 dye (Cy3)-conjugated, donkey, anti-rabbit IgG-specific antisera. The sections were then washed again (3 × 15 min) and coverslipped with carbonate-buffered glycerol (pH 8.6). Details concerning all the primary and secondary antibodies used are listed in Table 3.

The specificity of antisera was tested by standard controls, including pre-absorption of antibodies with the appropriate antigen, an omission test, and replacement of antisera by non-immune sera.

The total number of neurons containing CART and PGP 9.5 within submucosal and myenteric plexuses, as well as the total number of nerve fibers within the muscular layer in both tumour-affected and control areas of the human colonic wall were counted. The mean number (±SEM) of neurons inside the enteric plexuses was calculated in three randomly-chosen intestinal sections from each patient. These sections were separated by at least 400 µm to avoid double counting of the same neuronal structures. The population of CART and PGP 9.5-immunoreactive neuronal cells was subdivided into two subpopulations containing simultaneously CART and PGP 9.5, or devoid of CART but containing PGP 9.5. The number of neurons expressing PGP 9.5 in each “kind” of plexus (Table 2) was considered as the total cells number (100%). The percentage of neurons in each subpopulation was calculated.

The total number of CART- and PGP 9.5-containing nerve fibers in the MM, CM and LM were counted using the Merz grid from Fiji software [52]. The grid consists of a square that limits the test area, containing a system of points marked on a sinuous line. The Merz grid can be used to count points on a histological structure, which cuts the curved line. The results were then converted to the number of nerve fibers per unit area (µm^2^).

The immunofluorescence of neurons and nerve fibers was analysed using an Olympus BX61 microscope (Olympus, Tokyo, Japan) equipped with epi-fluorescence and appropriate filter sets for AMCA (V1 module, excitation range 330–385 nm, and barrier filter at 420 nm) and CY3 (G1, excitation filter 510–550 nm). Images were captured with a digital camera (Olympus U-LH100HG, Tokyo, Japan) and analysed with Cell Sens Dimension image-analysing software (Olympus, Tokyo, Japan).

### 4.4. Statistical Analysis

The non-parametric Mann-Whitney *U*-test was performed to evaluate differences in the distribution of CART in neurons of enteric plexuses and nerve fibers derived from the vicinity to cancer invasion and the control part of the colonic wall. The Kruskal-Wallis ANOVA and median test, with multiple post-hoc comparisons of mean ranks, was used to estimate the differences in the distribution of CART in neurons of each ganglionated plexus and nerve fibers located in the MM, CM and LM. The ANOVA Tukey test was prepared to compare the total number of PGP 9.5-containing neurons in the MP, OSP and ISP. Data are presented as means ± SEM. All statistical calculations were performed using Statistica (StatSoft Inc., Tulsa, OK, USA). The differences were considered to be significant at *p* < 0.05.

## Figures and Tables

**Table 1:** The total number of protein gene product (PGP) 9.5-containing neurons in the myenteric plexus (MP), outer submucosal plexus (OSP), and inner submucosal plexus (ISP) in control and cancer-affected areas of the human colonic wall. Data are presented as means ± SEM.

“Kind” of Ganglionated Plexuses	Control Tissue from Operative Margin	Cancer Affected Area	*p* Value
MP	98.7 ± 18.0	73.4 ± 15.3	0.56
OSP	12.0 ± 6.7 *	12.5 ± 5.6	0.83
ISP	24.7 ± 6.9 **	28.4 ± 9.4	0.68

Data are presented as means (±SEM) of the number of nerve fibers per area unit (µm^2^); * indicates differences (*p* < 0.01) between the MP and OSP from control tissue; ** indicates differences (*p* < 0.05) between the MP and ISP from control tissue.

**Table 2:** The number of CART-LI nerve fibers in the longitudinal muscle layer (LM), circular muscle layer (CM) and lamina muscularis mucosa (MM) in control and cancer-affected areas of the human colonic wall.

Muscle Layer of Intestinal Wall	Control Tissue from Operative Margin	Cancer Affected Area
LM	0.011 ± 0.002	0.014 ± 0.002
CM	0.031 ± 0.004 ^a^	0.036 ± 0.006 ^b^
MM	0.046 ± 0.014 ^a^	0.034 ± 0.008

Data are presented as means (±SEM) of the number of nerve fibers per area unit (µm^2^); ^a^ indicates differences (*p* < 0.05) between the LM and CM as well as MM from control tissue; ^b^ indicates differences (*p* < 0.01) between the LM and CM from cancer tissue.

**Table 3:** Characteristic of the antibodies used for double-immunofluorescence staining.

Antibody	Species	Catalog Number	Company	Dilution
Primary antibody				
CART (61–102)	rabbit	H-003-61	Phoenix Pharmaceuticals, Inc., Burlingame, CA, USA	1:6000
PGP 9.5	mouse	7863-2004	Biogenesis, Kingstone, NH, USA	1:2100
Secondary antibody				
Polyclonal Goat Anti-Rabbit Immunoglobulins/Biotinylated		E0432	Dako, Glostrup, DK	1:1000
AMCA-conjugated donkey anti-mouse IgG		715-156-151	Jackson Immunoresearch, West Grove, PA, USA	1:90
Cy™3-conjugated streptavidin		016-160-084	Jackson Immunoresearch, West Grove, PA, USA	1:9000

Abbreviations: CART—cocaine- and amphetamine-regulated transcript; PGP 9.5—protein gene product 9.5; AMCA—aminomethylcoumarin.
